# Establishment of an *Agrobacterium tumefaciens*-Mediated Transformation System for *Hirsutella sinensis*

**DOI:** 10.3390/cimb46090629

**Published:** 2024-09-22

**Authors:** Lijuan Wu, Xinkun Hu, Shen Yan, Zenglin Wu, Xuzhong Tang, Lei Xie, Yujie Qiu, Rui Li, Ji Chen, Mengliang Tian

**Affiliations:** 1 College of Agronomy, Sichuan Agricultural University, Chengdu 611130, China; wulijuan0921@126.com (L.W.); 17683180748@163.com (L.X.); 71352@sicau.edu.cn (R.L.); 2 Institute of Ecology, China West Normal University, Nanchong 637009, China; 3 State Key Laboratory of Crop Gene Resources and Breeding, Institute of Crop Sciences, Chinese Academy of Agricultural Sciences, Beijing 100081, China

**Keywords:** *Ophiocordyceps sinensis*, *Hepialidae*, *Hirsutella sinensis*, *Agrobacterium tumefaciens*-mediated transformation (ATMT), green fluorescent protein (GFP)

## Abstract

*Ophiocordyceps sinensis* (Berk.) is a complex is formed by *Hepialidae* larvae and *Hirsutella sinensis*. Infestation by *H. sinensis*, interaction with host larvae, and fruiting body development are three crucial processes affecting the formation of *O. sinensis*. However, research on the molecular mechanism of *O. sinensis* formation has been hindered by the lack of effective genetic transformation protocols. Therefore, *Agrobacterium tumefaciens*-mediated transformation (ATMT) was adopted to genetically transform two *H. sinensis* strains and optimize the transformation conditions. The results revealed that the most suitable *Agrobacterium* strain for *H. sinensis* transformation was AGL1, and that the surfactant Triton X-100 could also induce ATMT, although less effectively than acetosyringone (AS). In addition, the endogenous promoters of *H. sinensis* genes had a stronger ability to drive the expression of the target gene than did the exogenous promoter. The optimal transformation conditions were as follows: AS and hygromycin B concentrations of 100 μM and 50 μg/mL, respectively; *A. tumefaciens* OD_600_ of 0.4; cocultivation at 18 °C for 24 h; and *H. sinensis* used within three passages. The results lay a foundation for the functional study of key regulatory genes involved in the formation of *O. sinensis*.

## 1. Introduction

*Ophiocordyceps sinensis* (Berk.), a complex of stroma and larval corpse, is formed by the fungus of *Hirsutella sinensis* invading and parasitizing in the larva of Hepialidae insect. *O. sinensis* is valuable in traditional Chinese medicine due to the variety of effects it has on health, including tonifying effects on kidneys and lungs, blood coagulation, and phlegm, among others. *O. sinensis* has been used for medicinal purposes in China for centuries, but to date, the field has relied mainly on the use of wild resources. Although some progress has been made in the artificial cultivation of *O. sinensis*, it is still difficult to develop and expand the scale of artificial cultivation due to the lack of knowledge regarding the interaction mechanism between *H. sinensis* and Hepialidae larvae [1] and the poor understanding of the genetic mechanism of *H*. *sinensis* fruiting body development. These problems need to be addressed via further research.

*H. sinensis* was previously identified as the asexual form of *O. sinensis* [2]. Genome sequencing of *H. sinensis* has been completed in recent years, and its genome data are publicly available [3,4,5,6]. To date, genome-wide identification of MAPK (Mitogen-activated protein kinase), MAPKK (Mitogen-activated protein kinase kinase), MAPKKK (Mitogen-activated protein kinase kinase kinase), and other gene families has been performed on the basis of these genomic data, and correlation analysis of the haplotype of the NADPH-cytochrome P450 reductase gene and infection ability has been performed [7,8]. These findings suggest that the genomic data of *H. sinensis* are helpful for studying the genetic regulatory mechanism underlying its growth and development. By creating *H. sinensis* mutants, the genome sequence information could be changed to obtain different genotypes and produce individuals with different phenotypes to study gene function. To date, methods for the preparation of protoplasts from *H. sinensis* have been reported [9], as has the construction of a mutant using a plasma (ARTP) jet combined with ethyl methylsulfonate (EMS) for mutagenesis of protoplasts in *H. sinensis* [10]. However, these methods rely on protoplasts as the receptor and have many limitations that restrict their application; for example, protoplast culture is difficult and has a low regeneration rate, a high contamination rate, and complex operating procedures. In addition, the number of obtained mutants is low and the direction of mutation is uncertain. Therefore, it is necessary to develop a new, convenient, and efficient genetic transformation method to create *H. sinensis* mutants.

The *A. tumefaciens*-mediated transformation (ATMT) method has been effectively applied to create mutants of various filamentous fungi [11,12,13,14,15]. Compared with the PEG (Polyethylene glycol)-mediated transformation method, the ATMT method, with the advantages of simple operation, high transformation efficiency, good repeatability, and ability to achieve random knock-in of target genes and generate knock-in mutations [16], can be used to randomly insert T-DNA into the genome as a vector [17]. Therefore, this study aimed to establish a genetic transformation system for *H. sinensis* via the ATMT method and to optimize the transformation conditions. At present, mycelia, protoplasts, and conidia are commonly used as receptor materials for the genetic transformation of filamentous fungi. However, our previous study revealed that the sporulation rate of the *H. sinensis* strains KK_1_ and LT_4_ isolated from wild *O. sinensis* resources was notably low, with KK_1_ having a sporulation rate of only 5.6 × 10^9^ cfu/g and a spore germination rate of approximately 1%, whereas LT_4_ exhibited almost no sporulation. Additionally, previous studies reported that *H. sinensis* has the disadvantages of slow growth, a low sporulation rate, and a low spore germination rate [18,19], making it unsuitable for use as a receptor material. In contrast, mycelia are easy to obtain, simple to culture, and easy to manipulate under laboratory conditions [20]. Therefore, *H. sinensis* mycelia in liquid culture were used as receptor materials for the study of ATMT in this study. Finally, the *GFP* reporter gene and *hygromycin B* selection gene were successfully introduced into the genomic DNA of *H. sinensis* via random insertion of T-DNA and expressed, confirming the feasibility of this method for the genetic transformation of *H. sinensis*.

## 2. Materials and Methods

### 2.1. Experimental Strains

The experimental *H. sinensis* strains KK_1_ and LT_4_ were isolated from wild *O. sinensis* resources in Kangding and Litang Counties, Sichuan, China, respectively. They were deposited at the China General Microbiological Culture Collection Center (CGMCC) under the deposit numbers CGMCC NO. 41330 and CGMCC NO. 41331. The *A. tumefaciens* strains used in this study included GV3101, LBA4404, EHA105, and AGL1.

### 2.2. Culture Conditions

The ATMT method for *Aspergillus flavus* [12] was adopted for *H. sinensis* genetic transformation with appropriate modifications. The strains KK_1_ and LT_4_ were inoculated in BM liquid medium (containing 5 g/L tryptone, 10 g/L yeast extract, 30 g/L glucose, 1 g/L KH_2_PO_4_, and 0.5 g/L MgSO_4_) and incubated in darkness at 18 °C and 120 r/min for 7 days. A single colony of *A. tumefaciens* was inoculated into 2 mL of LB liquid medium containing 50 μg/mL kanamycin and 20 μg/mL rifampicin. After shaking at 28 °C and 180 r/min for 2 days, 1 mL of the culture was incubated in 25 mL of fresh LB liquid medium at 28 °C and 180 r/min for 6 h. After the prepared *A. tumefaciens* culture was mixed with *H. sinensis* mycelial suspension at a volume ratio of 1:3, 200 μL of the mixture was spread onto coculture BM (CO-BM) medium supplemented with 100 μM acetosyringone (AS), followed by coculturing at 18 °C for 24 h. The cocultured mycelium pellets were subsequently transferred to BM medium supplemented with 50 μg/mL hygromycin B and incubated in darkness at 18 °C for 30 days, until transformants emerged. The number of colonies on five plates was counted, and the transformation efficiency was calculated by the ratio of the number of counted colonies to the number of originally transferred mycelium pellets, and this method was also applicable to subsequent sections. The transformants were subsequently transferred to fresh BM plates containing 25 μg/mL hygromycin B for 30 days for further selection and verification, and the experiment was repeated three times.

### 2.3. Screening of A. tumefaciens Strains and Inducers

The pCAMBIA2300-GFP vectors were transformed into *A. tumefaciens* strains GV3101 (genotype: C58 (rif^R^) Ti pMP90 (pTiC58DT-DNA) (gent^R^) Nopaline pSoup (tet^R^)), LBA4404 (genotype: Ach5 (rif^R^) Ti pAL4404 (strep^R^) Octopine pSoup (tet^R^)), EHA105 (genotype: C58 (rif^R^) Ti pEHA105 (pTiBo542DT-DNA) Succinamopine pSoup (tet^R^)), and AGL1 (genotype: C58 RecA (rif^R^/carb^R^) Ti pTiBo542DT-DNA Succinamopine pSoup (tet^R^)). The methods for preparing and cocultivating the *A. tumefaciens* suspension and *H. sinensis* mycelia were the same as those described above. First, the classic inducer AS was used for induction at a concentration of 100 μM, and five replicate experiments were conducted. The GFP fluorescence intensity was observed in the *H. sinensis* mycelia to identify the most appropriate *A. tumefaciens* strain for infecting *H. sinensis*. The screened *A. tumefaciens* strain was subsequently used to prepare a bacterial suspension, which was cocultured with the prepared *H. sinensis* mycelia and then induced by adding AS or Triton X-100 at concentrations of 0, 10, or 100 μM. The experiment was repeated five times. By observing the fluorescence intensity of GFP using a fluorescence microscopy (Carl Zeiss AG, Oberkochen, Germany), the most suitable inducer and its optimal concentration were selected. The mean gray value of fluorescent graphs is used to represent the fluorescence intensity, and is calculated using the ImageJ (v1.8.0) software [21] with the equation “mean gray value (Mean) = integrated density of the region (IntDen)/area of the region (Area)”.

### 2.4. Screening of Promoters

The promoter sequences of the ribosomal protein S30 (30S)-, histone H3 (His3)-, and heat shock 70 kDa protein (HSP70)-encoding genes, which are highly expressed at all growth stages, were identified from the genome data of the *H. sinensis* KK_1_ strain via local BLAST analysis. Then, primers were designed according to these sequences (Table S1), and PCR amplification and gene cloning were performed using the genomic DNA of the KK_1_ strain as a template. The 35S promoter in the pCAMBIA2300 binary vector was subsequently replaced via homologous recombination, and expression vectors carrying the promoters of the *30S*, *His3*, and *HSP70* genes were constructed. The methods used for the transformation of these expression vectors into the screened optimal *A. tumefaciens* strains and for cocultivation were the same as above. The GFP fluorescence intensity was observed in the *H. sinensis* mycelia to screen for the promoter that initiated GFP expression most effectively.

### 2.5. Optimizing ATMT for H. Sinensis

To determine the minimum concentration required for effective inhibition of *H. sinensis* growth in the ATMT experiments, three concentrations of hygromycin B, i.e., 50, 100, and 150 μg/mL, were used for testing the effects of hygromycin B (Coolaber, Beijing, China) on the growth of *H. sinensis*. The transgenic hyphae were initially cultured on BM liquid medium for 7 days and then transferred to BM solid medium containing various concentrations of hygromycin B and incubated in darkness at 18 °C for 30 days. Then, the mycelia were quantified.

In accordance with the above genetic transformation method, the solution of *A. tumefaciens* was mixed with the mycelia of *H. sinensis* strains KK_1_ and LT_4_ and then spread on CO-BM plates. Each transformation event was repeated five times, and the transformation efficiency was determined after cocultivation at 18 °C for 24 h and 48 h and then after incubation in darkness at 18 °C for 30 days (until transformants emerged) to identify the optimal cocultivation time. Moreover, the suspension of *A. tumefaciens* was mixed with the mycelia of *H. sinensis* strain KK_1_ and spread on CO-BM plates. The cocultivation was carried out at 12 °C and 18 °C, with five replicate experiments at each temperature. The transformation efficiency was determined after cocultivation at 12 °C or 18 °C for 24 h, 48 h, and 60 h and after incubation in darkness at 18 °C for 30 days to identify the optimal cultivation temperature. Additionally, the OD_600_ values of the *A. tumefaciens* cultures were adjusted to 0.4, 0.6, and 0.8 by resuspending the pellets of centrifugation in BM liquid medium, respectively, and the cultures were then mixed directly with the mycelia of *H. sinensis* strain KK_1_ at these concentrations. The other genetic transformation steps were the same as those described above. Each concentration was tested with five replicate experiments, and the transformation efficiency was monitored after coculturing for 24 h and incubation in darkness at 18 °C for 30 days to identify the optimal *A. tumefaciens* concentration. The suspension of *A. tumefaciens* was mixed with the mycelia of the *H. sinensis* strain KK_1_, which had been passaged three and five times, and then spread on CO-BM plates. The other genetic transformation steps were the same as those described above, and each transformation event was repeated five times. The transformation efficiency was determined after coculturing at 18 °C for 24 h and incubation in darkness at 18 °C for 30 days to identify the optimal number of passages for *H. sinensis*.

### 2.6. PCR Analysis of Transformants

The genomic DNA of the *H. sinensis* wild-type strains KK_1_ and LT_4_ and the corresponding eight randomly selected transformants was extracted via the cetyltrimethylammonium bromide (CTAB) method. The empty plasmid DNA and the genomic DNA of the wild-type strain were used as positive and negative controls, respectively. PCR amplification was performed via *Hyg*-specific primers. The total volume of the PCR mixture was 50 μL, which included 25 μL of Tap Mix (Vazyme Biotech, Nanjing, China), 50 ng of template DNA, 1 μL of each primer (10 μM), and ddH_2_O to 50 μL. The PCR program was as follows: predenaturation at 95 °C for 5 min; 30 cycles of 95 °C denaturation for 15 s, 60 °C annealing for 15 s, and 72 °C extension for 30 s; and a final incubation at 72 °C for 5 min. Then, 5 μL of the PCR products was examined via 1% agarose gel electrophoresis, and the positive PCR products were submitted to Beijing Tsingke Biotech Co., Ltd., for sequencing. Alignment analysis of the obtained nucleotide sequences was performed against the NCBI database (https://www.ncbi.nlm.nih.gov/ (accessed on 12 March 2023).

### 2.7. RNA Extraction and Gene Expression Analysis

The RNA of the transformants was extracted via the Spin Column Fungal Total RNA Purification Kit (Sangon Biotech, Shanghai, China), and 1 μg of total RNA was reverse transcribed via Evo M-MLV RT Premix for qPCR (Accurate Biology, Changsha, China). Both processes were performed according to the manufacturers’ instructions. RT–qPCR was conducted with the 10× diluted reverse transcription product as a template, with GFP as the test gene and β-actin as the internal reference gene. The primers GFP-qF and GFP-qR were utilized for this experiment (Table S1). Five microliters of each 10× diluted cDNA was added to the 20 μL reaction mixture with SYBR Green Real-time PCR Master Mix (Accurate Biology, Changsha, China). RT–qPCR was carried out in a CFX Connect Real-time system (Bio-Rad, Foster, CA, USA) as follows: 5 min at 95 °C; 40 cycles of 30 s at 95 °C, 30 s at 60 °C, and 30 s at 72 °C; melting curve analysis was conducted from 55 °C to 95 °C, reading every 1 °C and holding for 5 s. Two technical replicates for each sample and three biological replicates were carried out. The relative expression levels were calculated via the 2^−ΔΔCt^ method [22].

### 2.8. Detection of the Genetic Stability of Transformants

A vector with a 30S promoter driving *GFP* expression and an HSP70 promoter driving *HygB* expression was constructed on the basis of the binary vector pCAMBIA2300. After *H. sinensis* was transformed with the vector, ten transformants were randomly selected and cultured on BM. After incubation for 30 days, hyphae from the colony periphery were picked and subcultured on fresh BM. The cultures were continuously passaged and cultured for five generations and then placed under a fluorescence microscope to observe the expression of GFP and to confirm the genetic stability of the transformed strain.

### 2.9. Statistical Analysis

Statistical analysis was carried out using SPSS version 19.0 for analysis of variance (ANOVA), followed by Duncan’s multiple range test (DMRT) to compare means among treatments if the ANOVA result was significant (*p* ≤ 0.05). Before ANOVA, the data were tested for normality using Chi-squared analysis and transformed where necessary.

## 3. Results

### 3.1. Screening of A. tumefaciens Strains

**Figure 1:**
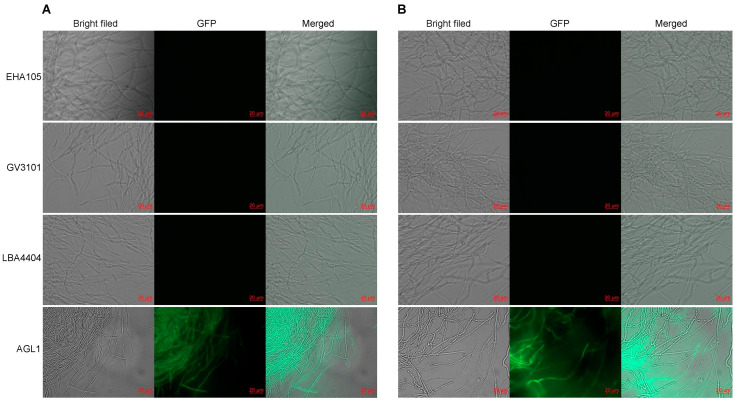
Infection by different *Agrobacterium tumefaciens* strains of two *Hirsutella sinensis strains*, KK_1_ (**A**) and LT_4_ (**B**).

Four *A. tumefaciens* strains with different genotypes, namely, EHA105, GV3101, LBA4404, and AGL1, were assessed for their ability to mediate the genetic transformation of *H. sinensis* KK_1_ and LT_4_. Empty pCAMBIA2300-35S-GFP vector was separately transformed into each of the above four *A. tumefaciens* strains, and the transformants were used to infect *H. sinensis* strains KK_1_ and LT_4_. Then, the transient expression of GFP was monitored. The results revealed that only the AGL1 strain exhibited green fluorescence after infecting *H. sinensis*, indicating that *H. sinensis* had been successfully infected by AGL1 and that GFP was expressed (Figure 1).

### 3.2. Effect of Inducers on Transformation Efficiency

**Figure 2:**
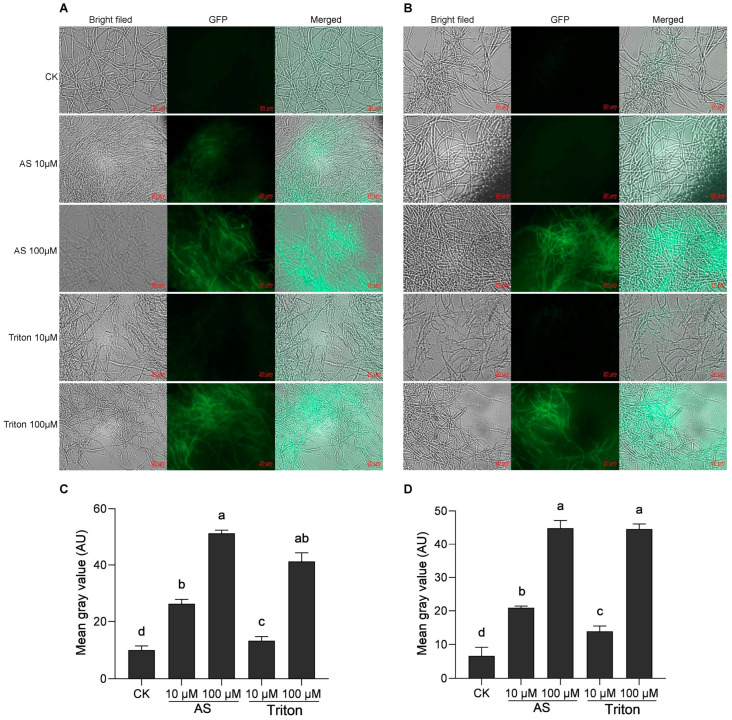
Induction by two different inducers (AS and Triton X-100) in the mixture of *Agrobacterium* and two *H. sinensis* strains, KK_1_ (**A**) and LT_4_ (**B**). Significance analysis of the mean gray value of the GFP graphs in strains KK_1_ (**C**) and LT_4_ (**D**). AU, arbitrary unit. The letters a–d above bar indicated significant difference at *p* < 0.05 level.

Acetosyringone is a commonly used inducer and is very important for ATMT experiments. In this study, *A. tumefaciens* was induced with 10 μM and 100 μM AS and was simultaneously induced with the surfactant Triton X-100 at 10 μM and 100 μM, respectively. Analysis of the transient expression of GFP revealed that 48 h after transformation, the green fluorescence of GFP could be detected under both induction and simulated induction conditions. The intensity of the green fluorescence gradually increased with increasing concentrations of AS and Triton X-100, and the number of luminous mycelia increased simultaneously (Figure 2A,B). When *Agrobacterium* was induced by 10 μM AS, the green fluorescence signal of *H. sinensis* was significantly stronger than that induced by 10 μM Triton X-100 (Figure 2C,D). However, there was no significant difference when induced by 100 μM AS and Triton X-100, with the former slightly stronger than the latter.

### 3.3. Optimization of Promoters

**Figure 3:**
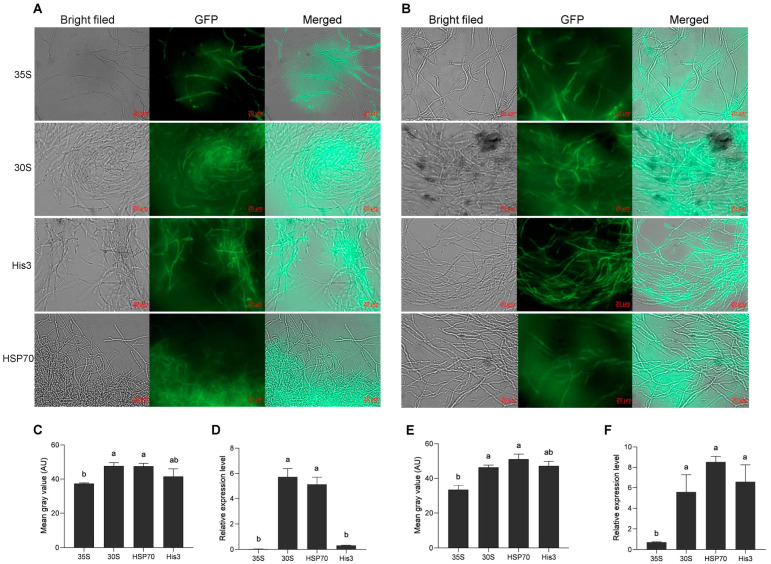
Effects of different promoters on GFP expression in two *H. sinensis* strains, KK_1_ (**A**) and LT_4_ (**B**). Significance analysis of the relative expression levels of GFP driven by different promoters in strains KK_1_ (**C**) and LT_4_ (**E**). Significance analysis of the mean gray value of the GFP graphs in strains KK_1_ (**D**) and LT_4_ (**F**). AU, arbitrary unit. The letters a and b above bar indicated significant difference at *p* < 0.05 level.

The promoter sequences of the *30s*, *His3*, and *HSP70* genes were amplified from KK_1_ genomic DNA. The pCAMBIA2300-30S-GFP, pCAMBIA2300-His3-GFP, and pCAMBIA2300-HSP70-GFP plasmids were constructed on the basis of the pCAMBIA2300-35s-GFP vector and were subsequently transformed into *A. tumefaciens* strains AGL1. The *H. sinensis* strains KK_1_ and LT_4_ were subsequently infected separately with positive *A. tumefaciens* strains carrying the above three plasmids, and the green fluorescence of GFP was detected in all of them (Figure 3A,B), confirming that the above three promoters could activate the expression of GFP. Additionally, the relative expression level of GFP activated by the *His3* promoter in the KK_1_ strain was not significantly different from that activated by 35S, but the relative expression level of GFP activated by the promoters of the *30s* and *HSP70* genes significantly differed from that activated by 35S (Figure 3C). The relative expression levels of GFP activated by the three promoters in the LT_4_ strains were significantly greater than those activated by 35S (Figure 3E). In both KK_1_ and LT_4_ strains, the mean gray values of the graph of GFP activated by the promoter of the *30s* and *HSP70* gene were significantly different from those activated by the 35S (Figure 3D,F).

### 3.4. Sensitivity of H. sinensis to the Hygromycin B Concentration

**Figure 4:**
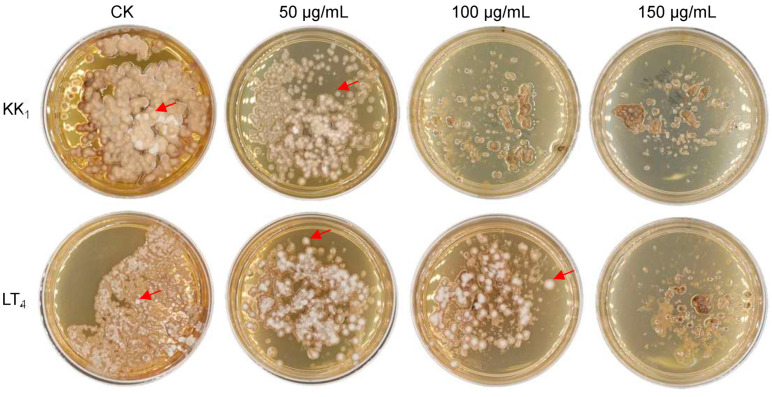
Sensitivity of two *H. sinensis* strains, KK_1_ and LT_4_, to hygromycin B. The red arrows indicated the colonies of *H. sinensis* strains KK_1_ and LT_4_.

The mycelia of *H. sinensis* strains KK_1_ and LT_4_ were inoculated on BM medium supplemented with 0 μg/mL, 50 μg/mL, 100 μg/mL or 150 μg/mL hygromycin B. The growth of the two strains was significantly inhibited on medium supplemented with 50 μg/mL hygromycin B after 30 days of static culture. In addition, strain KK_1_ could not grow on media supplemented with more than 100 μg/mL hygromycin B. However, strain LT_4_ produced a small number of colonies on medium supplemented with 100 μg/mL hygromycin B, but the growth was completely inhibited on medium supplemented with 150 μg/mL hygromycin B (Figure 4).

### 3.5. Optimization of ATMT Conditions

#### 3.5.1. Effect of Coculture Time on Transformation Efficiency

**Figure 5:**
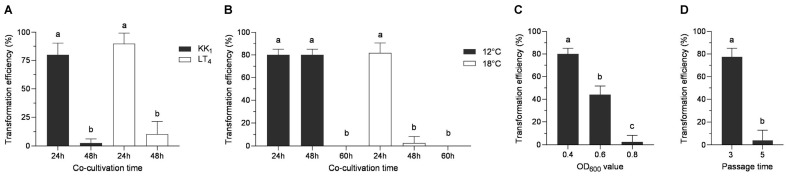
Effects of different factors on the transformation efficiency of *H. sinensis*. These factors included the cocultivation time (**A**), cocultivation temperature (**B**), cell concentration of *A. tumefaciens* (**C**), and number of mycelial passages (**D**). The letters a–c above bar indicated significant difference at *p* < 0.05 level.

The pCAMBIA2300-35S-GFP vector was modified by replacing the 35S promoter and the *GFP* gene with the *30S* gene promoter and the *Hyg* gene, respectively, and the pCAMBIA2300-30S-Hyg plasmid was thus obtained. Then, the plasmid was transformed into *H. sinensis* strains KK_1_ and LT_4_, and the strains were then cocultured at 18 °C for 24 h or 48 h. After screening on BM medium supplemented with 50 μg/mL hygromycin B, the two strains presented the greatest number of transformants, and the transformation efficiency reached 80% or more when the coculture time was 24 h, whereas the number of transformants obtained was very low, and the transformation efficiency was less than 10%, when the coculture time was 48 h (Figure 5A).

#### 3.5.2. Effect of Coculture Temperature on Transformation Efficiency

The optimal culture temperature for *H. sinensis* was 18 °C, whereas the optimal growth temperature for *A. tumefaciens* was 28 °C. *H. sinensis* was severely impaired when the transformation temperature was too high, while too low of a transformation temperature caused a severe reduction in the transformation efficiency of *A. tumefaciens*. In this study, the pCAMBIA2300-30s-Hyg plasmid was transformed into strain AGL1 and cocultured with strain KK_1_. The transformation efficiency was analyzed at two temperatures (12 °C and 18 °C) and three time points (24, 48, and 60 h). The results revealed that the transformation efficiency was approximately 80% when the coculture temperature was 12 °C and the duration was 24 h or 48 h, and the transformation efficiency decreased to zero as the coculture time increased to 60 h. When the coculture temperature was 18 °C, the transformation efficiency was approximately 80% at 24 h and decreased to 2.6% as the time increased to 48 h. The transformation efficiency decreased to zero when the coculture time increased to 60 h (Figure 5B).

#### 3.5.3. Effect of *A. tumefaciens* Concentration on Transformation Efficiency

The concentration of *A. tumefaciens* plays a crucial role in fungal genetic transformation. In this study, AGL1 suspensions with OD_600_ values of 0.4, 0.6, and 0.8 were mixed with KK_1_ suspensions at a volume ratio of 1:3. After being cocultured at 18 °C for 24 h, the transformation efficiency reached 80% when the optical density of the AGL1 suspension was 0.4, whereas the transformation efficiency was as low as zero when the optical density of the AGL1 suspension was 0.8 (Figure 5C).

#### 3.5.4. Effect of Mycelial Passage Time on Transformation Efficiency

The more times mycelia are subcultured, the older and less robust they become. In this study, mycelia subcultured three or five times were used for coculture with *A. tumefaciens*, and the results revealed that the transformation efficiency was the highest when the number of passages was three, reaching 87%. However, the transformation efficiency decreased to nearly zero for the mycelia that were subcultured five times (Figure 5D).

### 3.6. Stability of Transformants

**Figure 6:**
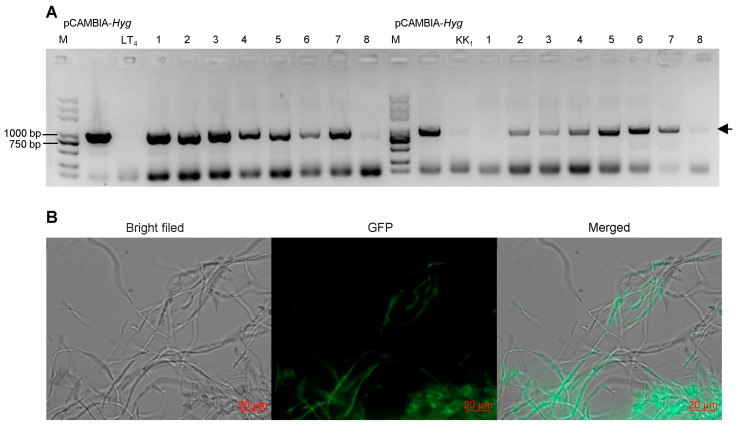
PCR detection (**A**) and fluorescence observation (**B**) of transformants. The arrowhead indicated the target band.

Eight positive transformants were randomly selected from strains LT_4_ and KK_1_ to extract genomic DNA, and PCR was used to amplify the target band of the *Hyg* gene. The electrophoresis results revealed that seven positive LT_4_ transformants and six positive KK_1_ transformants produced the target band (Figure 6A). The six positive KK_1_ transformants were subsequently selected for culture on basic medium. After 30 days of cultivation, the mycelial pieces on the edge of the colony were transferred to fresh basic medium for culture and then transferred to the selection medium containing 25 μg/mL hygromycin B after three consecutive subcultures. The results showed that these six transformant strains could still grow normally. The results confirmed that the exogenous T-DNA structure was successfully inserted into the strain KK_1_ genome and could be stably inherited (Figure 6B).

## 4. Discussion

### 4.1. Establishment of ATMT for H. sinensis

At present, genetic transformation systems for fungi are mainly established via ATMT-mediated and PEG-mediated methods. PEG-mediated genetic transformation uses protoplasts as the receptor material. For example, previous studies have successfully established a genetic transformation system for *Fusarium oxysporum* [23] and *Verticillium dahliae* [24] via the PEG-mediated protoplast transformation method. However, the process of protoplast transformation is complicated, and the preparation process can be influenced by mycelial age and enzyme concentration. The process has the disadvantages of a low regeneration rate and ease of contamination. In contrast, the ATMT method allows the direct use of mycelia as the receptor material, eliminating the need for protoplast preparation, and has the advantages of easy operation and high transformation efficiency and stability. Therefore, in this study, mycelial fragments were used as the receptor material to screen and optimize the parameters in the ATMT system of *H. sinensis*, such as *A. tumefaciens* strains, inducers, promoters, hygromycin B concentrations, coculture time and temperatures, *A. tumefaciens* concentrations, and mycelial age. Finally, a stable and efficient ATMT system for *H. sinensis* independent of protoplasts was successfully established.

### 4.2. Effects of Different A. tumefaciens Strains on ATMT in H. sinensis

The ATMT method has been widely used in the genetic transformation of filamentous fungi. However, previous studies have shown that there are significant differences in the infection abilities of various *A. tumefaciens* strains when they are applied to different fungal species. For example, the effects of three *A. tumefaciens* strains on the genetic transformation of *Trichoderma* spp. have been studied, revealing that strains AGL-1 and GV3101 could successfully mediate genetic transformation, with the former having a slightly greater transformation efficiency than the latter, whereas strain LBA4404 was unable to mediate genetic transformation [25]. In contrast, the LBA4404 strain was better at mediating the genetic transformation of *Penicillium chrysogenum* [26]. Therefore, *A. tumefaciens* strains for use in ATMT for *H. sinensis* were screened in this study, which revealed that, among the four strains, only AGL1 exhibited the green fluorescence of GFP after transformation, indicating that it could mediate the genetic transformation of *H. sinensis*. The results also confirmed that *A. tumefaciens* strains have host specificity in mediating fungal genetic transformation.

### 4.3. Effects of Inducer Type and Concentration on ATMT in H. sinensis

AS is a phenolic substance that induces the ATMT process and plays an important role in the successful insertion of T-DNA fragments into the receptor into the recipient genome. Previous studies have shown that an AS concentration ranging from 0 to 200 μM is positively correlated with transformation efficiency and that a concentration of 200 μM had the best induction effect and the highest transformation efficiency, while the transformation efficiency decreased significantly when the concentration of AS reached 300 μM [27,28]. In this study, two *H. sinensis* strains were induced and simulated with AS and Triton X-100 at two different concentrations. The results revealed that the green fluorescence of GFP could be detected in both cases, but the induction effect of AS was significantly better than that of Triton X-100 (Figure 2). The study also revealed that the concentration of AS was positively correlated with the transformation efficiency, which was consistent with the results of previous studies.

Triton X-100 is a nonionic surfactant that can dissolve lipids and increase cell membrane permeability, damaging cells. Under Triton X-100 stress, fungal cells produce many phenolic substances to cope with the stress [29]. Additionally, the effects of Triton X-100 on the production of laccase and degradation of phenol by *Penicillium simplicissimum* in solid-state fermentation have previously been studied, revealing that Triton X-100 had a certain inhibitory effect on the production of laccase by *P. simplicissimum* and that the inhibitory effect became stronger with increasing concentration [30]. In this study, the green fluorescence of GFP was detected under simulated induction with Triton X-100, indicating that *A. tumefaciens* also successfully inserted T-DNA fragments into the receptor strain. The possible reason was that Triton X-100 treatment inhibited the production of laccase in *H. sinensis*, resulting in the production of phenolic substances, thus activating the *A. tumefaciens* Vir region and promoting the insertion of T-DNA fragments. With increasing Triton X-100 concentration, the inhibitory effect on laccase production increased, and the concentration of phenolic substances also increased, resulting in a simultaneous improvement in transformation efficiency (Figure 2).

### 4.4. Analysis of the Ability of Endogenous and Exogenous Promoters to Drive GFP Expression

*GFP* is a widely used reporter gene for genetic transformation and is famous for its fluorescence signal, which can be detected with high sensitivity and reliability. In previous studies, *GFP* was successfully introduced into *Valsa mali* var. *pyri* and *Verticillium dahliae*, resulting in the creation of T-DNA insertion mutants, and the fungal infection process was observed by GFP fluorescence [31]. Additionally, the GFP gene was efficiently transformed into *Colletotrichum gloeosporioides* via the PEG-CaCl_2_-mediated method, laying a foundation for observing the infection process of *C. gloeosporioides* on tea leaves via GFP [32]. Therefore, GFP can be used as a fluorescent label to screen positive transformants quickly and conveniently. However, the expression of GFP introduced by genetic transformation is usually driven by the exogenous cauliflower mosaic virus (CaMV) 35S promoter. The 35S promoter has been reported to affect not only the expression of downstream transgenes but also the expression of other nearby genes through its enhancer region [33,34]. Thus, an increasing number of researchers prefer to use endogenous promoters and UBQ10 promoters [35]. A comparison of the relative expression levels of *GFP* driven by three endogenous gene promoters of *H. sinensis* and the exogenous 35S promoter revealed that there was no significant difference in the relative expression of *GFP* driven by the promoters of His3 and 35S in the KK_1_ strain, whereas the relative expression of *GFP* driven by the promoters of three endogenous genes was significantly greater than the latter in other cases. The study confirmed that the endogenous promoters of *H. sinensis* were more suitable for the genetic transformation of its target gene than the exogenous 35S promoter.

### 4.5. Analysis of the Sensitivity of H. sinensis to Hygromycin B

Hygromycin B is an antibiotic used for selecting transgenic positive transformants, and the tolerance and sensitivity of hygromycin B differ among various fungi. For example, *Fusarium oxysporum* f. sp. *vasinfectum*, the pathogen that induces cotton Fusarium wilt, can tolerate hygromycin B concentrations as high as 300 μg/mL [36]. However, the mycelial growth of *Fusarium oxysporum* f. sp. *melongenae*, the pathogen causing eggplant Fusarium wilt, was completely inhibited when the concentration of hygromycin B was as high as 100 μg/mL [28]. The concentration of hygromycin B plays a crucial role in selecting positive transformants. Too high a concentration would affect the growth of transformants, while too low a concentration would increase the false-positive rate and the difficulty of selecting. In this study, the hygromycin B sensitivity of two *H. sinensis* strains, KK_1_ and LT_4,_ was studied. The growth of both strains was significantly inhibited when the hygromycin B concentration was 50 μg/mL. On the other hand, the mycelial growth of strain KK_1_ was completely inhibited when the concentration reached 100 μg/mL, while strain LT_4_ exhibited a small amount of colony growth at this concentration. Hence, the optimal hygromycin B concentration for selecting positive transformants of *H. sinensis* was 50 μg/mL.

### 4.6. Optimization of Other Factors Affecting ATMT in H. sinensis

In the ATMT system, the cocultivation time, temperature, and *A. tumefaciens* concentration play important roles in the genetic transformation efficiency for filamentous fungi. A previous study on the genetic transformation of *F. avenaceum* revealed that the transformation efficiency increased significantly as the cocultivation time increased from 24 h to 72 h and that the transformation efficiency increased continuously from 72 h to 96 h; but the difference was not significant [37]. However, it was reported that the transformation efficiency peaked at 96 h and 48 h after ATMT of *F. oxysporum* f. sp. *vasinfectum* and *Hypsizygus marmoreus*, respectively, and the transformation efficiency decreased with increasing cocultivation time [27,38]. In this study, the transformation efficiencies of the two *H. sinensis* strains were found to be highest at 24 h post-transformation and exceeded 80%. In contrast, the efficiencies decreased to less than 20% when the cocultivation time was extended to 48 h. The results also indicated that the transformation efficiencies decreased with increasing cocultivation time. This might be due to the excessive proliferation of *A. tumefaciens*, which caused the *H. sinensis* strains to stop growing under severe stress conditions. Therefore, the cocultivation time could be shortened to 24 h to improve the transformation efficiency.

In a study of ATMT of *H. marmoreus* and *Hebeloma cylindrosporum*, the optimal cocultivation temperatures were 26 °C and 23 °C, respectively, which are consistent with the optimal growth temperatures [39,40]. In contrast, a study on the genetic transformation of *Ganoderma lucidum* revealed that the optimal cocultivation temperature was 25 °C [41], which was different from the optimal growth temperature of 28 °C [42]. The optimal culture temperature for *H. sinensis* is 18 °C, and its mycelia grow slowly when cultured at 12 °C. Here, 12 °C and 18 °C were used as temperatures to explore the optimal temperature for ATMT of *H. sinensis*. The results showed that 12 °C was the most suitable cocultivation temperature, and a high transformation efficiency could be obtained at this temperature at both 24 h and 48 h. In contrast, the transformation efficiency was high only at 24 h when coculture was performed at 18 °C; if the time was extended to 48 h, *A. tumefaciens* could not be eluted from the mycelial surface due to excessive growth, resulting in a failure to screen *H. sinensis*.

Studies have shown that different concentrations of *A. tumefaciens* strains are required for the genetic transformation of different fungi. For example, previous studies indicated that the optimal OD_600_ value of *A. tumefaciens* for *Aspergillus flavus* genetic transformation was 0.3 [12], whereas that for *Dichomitus squalens* was 0.4 [43]. In this study, the transformation efficiency of *H. sinensis* was analyzed when the OD_600_ values of *A. tumefaciens* were 0.4, 0.6, and 0.8, and the findings showed that the optimal OD_600_ value for *A. tumefaciens* was 0.4. Hence, the concentration of *A. tumefaciens* should be adjusted to OD_600_ = 0.4 during the cocultivation for the genetic transformation of *H. sinensis*. Additionally, the number of hyphal subcultures is also an important factor affecting the efficiency of genetic transformation. The natural habitat for *O. sinensis* in China is the low-temperature and low-oxygen alpine meadow on the Qinghai–Tibet Plateau; it is easy for the fungus to age and degenerate in low-altitude areas with high oxygen levels but not in its natural habitat. The hyphae subcultured for three generations presented increased transformation efficiency, whereas the transformation efficiency of hyphae was close to zero after subculturing for five generations. Therefore, hyphae subcultured for three generations or less should be selected as the receptor material for ATMT of *H. sinensis*.

## 5. Conclusions

In this study, a stable ATMT-mediated genetic transformation system for *H. sinensis* was established. The results showed that AGL1 was the most effective *A. tumefaciens* strain for the transformation of *H. sinensis*. The genetic transformation efficiency was highest when the concentrations of AS and hygromycin B were 100 μM and 50 μg/mL, respectively; the OD_600_ value for *A. tumefaciens* was 0.4; the cocultivation temperature was 12 °C; the cocultivation time was 24 h; the number of passages of *H. sinensis* was less than three; and target gene expression was driven by the endogenous gene promoter from *H. sinensis*. Additionally, the surfactant Triton X-100 was also able to induce ATMT, but the effect was significantly weaker than that of AS. This study also successfully established GFP-labeled strains of *H. sinensis*, which laid a technical and material foundation for studying the functions of key regulatory genes involved in the interaction between *H. sinensis* and Hepialidae larvae, fruit body development, and pharmacological component biosynthesis.

## Data Availability

The original contributions presented in this study are available in the article or Supplementary Materials.

## Supplementary Materials

The following supporting information can be downloaded at https://www.mdpi.com/article/10.3390/cimb46090629/s1.
