# Supplementary material for: Establishment of an Agrobacterium tumefaciens-Mediated Transformation System for Hirsutella sinensis
Source: Curr Issues Mol Biol. 2024 Sep 22;46(9):10618–32. doi: 10.3390/cimb46090629 (PMC11430471; doi:10.3390/cimb46090629)
Supplement: Supplementary file 1 [file cimb-46-00629-s001.zip › cimb-3181659-supplementary.pdf]

Table S1. PCR primers used in this study.

Primers	Sequences (5´ to 3´)	Tm (°C)	Application
30S-F1	CCGGAATTCTTACTTGGTCGAGAGGGAGGGAATGAG	60	Promoter amplification
30S-R1	CCCGGATCCGTTCGAGTCCTGCGACGCTCCTTCCGGCT		
HSP70-F1	CCGGAATTCCGGAGATCCAAATCTTCTTCGACCTC	65	Promoter amplification
HSP70-R1	CCCGGATCCTTTCCTTGATACCCTCACTGTCCTGTC		
His3-F1	CCGGAATTCGGAACGTACAGAGTACCTTGATGTATGG	65	Promoter amplification
His3-R1	CCCGGATCCCGAAGCATTGGATGCCGCACACAGCGT		
HygB-F1	ATGAAAAAGCCTGAACTCACCGC	60	*HygB* amplification
HygB-R1	CTATTCCTTTGCCCTCGGAGGACG		
GFP-qF1	CGTAAACGGCCACAAGTTCA	60	RT-qPCR
GFP-qR1	CTTCATGTGGTCGGGGTAGC		
